# Morphological, physical, thermal, and mechanical properties with the aspect ratio effects of bio loose-fill packaging from corn stalk

**DOI:** 10.1038/s41598-023-41612-5

**Published:** 2023-09-12

**Authors:** Linda Thiraphattaraphun, Pattarapan Prasassarakich

**Affiliations:** 1 Division of Packaging Technology, Faculty of Agro-Industry, Chiang Mai University, Chiang Mai, 50100 Thailand; 2 Department of Chemical Technology, Faculty of Science, Chulalongkorn University, Bangkok, 10330 Thailand; 3 Center of Excellence on Petrochemical and Materials Technology, Chulalongkorn University, Bangkok, 10330 Thailand; 4 Center of Excellence in Green Materials for Industrial Application, Chulalongkorn University, Bangkok, 10330 Thailand

**Keywords:** Chemistry, Engineering, Materials science

## Abstract

Protective packaging, such as loose-fill material, is commonly used for void filling in packages during transportation and handling. Due to environment concerns about packaging materials, alternative materials derived from agricultural residues, such as corn stalks (CS), are of attention. Dried internodal CS without rind (DCS^-R^) were prepared as a cylindrical-shaped bio loose-fill packaging pieces (DCS^-R^P) at three different aspect ratios [length/diameter (L/D) of 0.4, 0.8, and 1.2]. The morphological, physical, and thermal properties of the DCS^-R^P were investigated and the effect of the L/D ratio of the DCS^-R^P was examined under compression loading. The DCS^-R^P exhibited a porous structure with a low density and bulk density, while the packing efficiency at all L/D ratios was less than 1. Different compressive resistance and failure patterns of the DCS^-R^P were obtained, depending on the direction of compression loading (parallel and perpendicular) to the DCS^-R^P. In addition, the L/D ratio of bulk DCS^-R^P also affected the compressive resistance. The results of this study provide important information for future investigations on the protective ability of DCS^-R^P to the products inside the packages during transportation and handling.

## Introduction

Corn stalks (CS) are an agricultural waste residue that is produced in large amounts after harvesting of corn crops and are typically used as animal feed, landfill, or burning. However, burning CS causes air pollution, especially PM2.5 (particulate matters of less than 2.5 µm), which can adversely affect peoples’ health. To increase the usage of agricultural residue, instead of burning it or using it in landfill, CS have been studied from both a scientific and technological point of view.

For example, the use of CS have been developed in composite technology for various applications, where it could be used as a reinforcing filler in thermoplastic composites, such as injection-molded CS waste/polypropylene composites, with improved tensile properties by using semichemical pulp rather than thermomechanical and chemical pulp from CS^1^. Moreover, CS-composite based superabsorbents prepared by graft-copolymerization of CS with acrylic acid, acrylamide, and sodium 4-styrenesulfonate could be applied for metal ion adsorption due to its high adsorption rates and relatively high adsorption capacities for Ni(II) and Cd(II) ions^2^. Interestingly, an alternative insulation composite made from CS mixed with epoxy resin exhibited low heat transfer coefficients (lower than 0.1 Wm^−1^ K^−1^)^3^. In addition, bio-composites for building insulation prepared by mixing CS and magnesium phosphate cement were found to have a good thermal conductivity and compressive strength^4^. Plant-concrete composites produced from pretreated CS particles and binder exhibited a decreased energy loss (40 to 60%) compared with a traditional brick wall in a heat loss analysis^5^. Recently, a solar steam generator made from a composite of CS, multi-walled carbon nanotubes, and titanium dioxide was applied for efficient desalination and gave an evaporation rate of 2.48 kg m^−2^ h^−1^ and an evaporation efficiency of 68.2% under solar light^6^.

For fuel and energy applications, CS pyrolysis gave a 30% yield of biochar that had kinetic thermal decomposition parameters (temperature range of 210–318 °C) of a mass loss of 52.6% and a weight active energy of 155.13 kJ mol^−1^^7^. Torrefaction as a pretreatment of CS pyrolysis could promote the generation of combustible gases and phenols of bio-oil^8^. However, the ignition temperature of torrefied CS was revealed to be between 251 and 296 °C and the burn-out temperature was limited at 520 °C^9^. In addition, a solvothermal liquefaction process (with ethanol as the solvent) of the CS to bio-oil products provided mostly phenolic compounds and their derivatives, while increasing the reaction temperature above 275 °C did not increase the bio-oil yield^10^.

For the development of particleboard and fiberboard from agricultural waste residue, the mechanical properties (modulus of rupture, modulus of elasticity, and internal bond) of a CS/woodchip particleboard was still lower than that of an industrial woodchip particleboard^11^. Similarly, the commercial fiberboard presented a superior internal bonding strength and impact strength than the CS/thermomechanical pulp fiberboard^12^. On the other hand, for binding applications, CS as a modifier in asphalt binders could improve the deformation resistance and elastic recovery of asphalt^13^.

Interestingly, the cellulose nanocrystals (a nano-filler in polymer) extracted from CS had a length/diameter (L/D) ratio of 18.9, width of 6.37 nm, length of 120.7 nm, crystallinity of 63.3% in a cellulose Iβ structure, and degradation temperature of 239.5 °C^14^. With respect to the mechanical properties of CS, the elastic moduli evaluated from three-point bending, and tensile and compression testing, were in the range of 6–16 GPa^15^. The failure patterns of CS in the bending analysis were revealed as three natural behaviors—crease, snap, and split^16^. In addition, during the three-point bending load, significant cross-sectional deformation occurred at the internodal sections of CS, whereas minimal cross-sectional deformation occurred at the nodal sections^17^. Moreover, the tensile properties of CS with rind (CS^+R^) exhibited an elastic–plastic behavior (linear at the initial and non-linear until rapture)^18^. In the compression test of CS^+R^, the breaking phenomena of the internodal section was ovalization, vertical break of the core, vertical break of the rind, horizontal break of the rind, and flattening, respectively^19^. Although, some researchers have investigated the compression loading of CS^+R^, to our knowledge, the behavior of CS without rind (CS^-R^) subjected to a compression load has never been reported.

For packaging applications, among biodegradable films developed from different sources of corn, such as corn starch^20^, corn zein^21^, and corn residues (CS, husks, and cobs)^22^, the use of CS as filler could improve the mechanical properties (tensile strength and Young’s modulus) of a corn starch film^22^. For logistic application, vibration, impact, or shock of product-to-product and product-to-package can occur during transportation and handling and result in damage to the products inside the packages. To reduce or negate such damage to products inside packages during transportation, protective packaging is employed to provide appropriate cushioning, void filling, block & bracing, and wrapping. Loose-fill packaging is commonly used in protective packaging and is typically composed of expandable polystyrene (EPS) filled in the box or bag for void filling. The loose-fill packaging is used to protect products of diverse shapes and to lock the product inside the package by filling the empty space between the product and package. However, there are increasing concerns on the environmental impact of non-degradable petroleum-based materials that are difficult to recycle, such as EPS. Thus, alternative choices of environmentally friendly loose-fill materials, such as starch and flour-based loose-fill^23,24^, cardboard loose-fill^23^, and wood shavings^25^, have been used as protective packaging materials.

The use of corn products for developing loose-fill packaging has mainly been based on corn starch^26,27^, whereas the use of other parts of corn have not been reported. Therefore, CS made from renewable resources could be an alternative choice for a bio loose-fill packaging. They can be formed as a tube shape, which is the commonly used shape of loose-fills, without any complex processing. Thus, the machinery cost and energy consumption of the production of dried CS^-R^ pieces (DCS^-R^P) could be lower than that for petroleum-based loose-fill packaging production. In addition, DCS^-R^P have not been used as a bio-loose-fill packaging. Therefore, the aim of this research was to prepare DCS^-R^P from internodal CS^-R^ as bio loose-fill packaging, and then to investigate their properties. In addition, the effect of the aspect ratio (length/diameter; (L/D) of the DCS^-R^P on the water absorption, moisture absorption, bulk density, packing efficiency, compressive properties, and failure pattern was evaluated.

## Experimental

### Materials and loose-fill preparation

**Figure 1:**
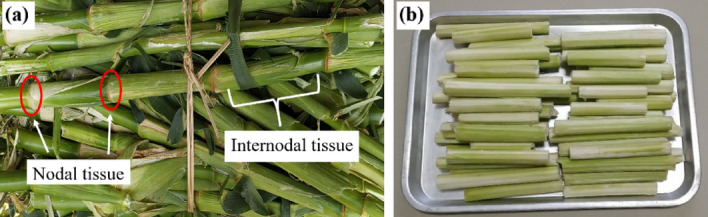
Appearance of the (**a**) fresh CS^+R^ and (**b**) internodal CS^-R^ before drying in a hot air oven.

**Figure 2:**
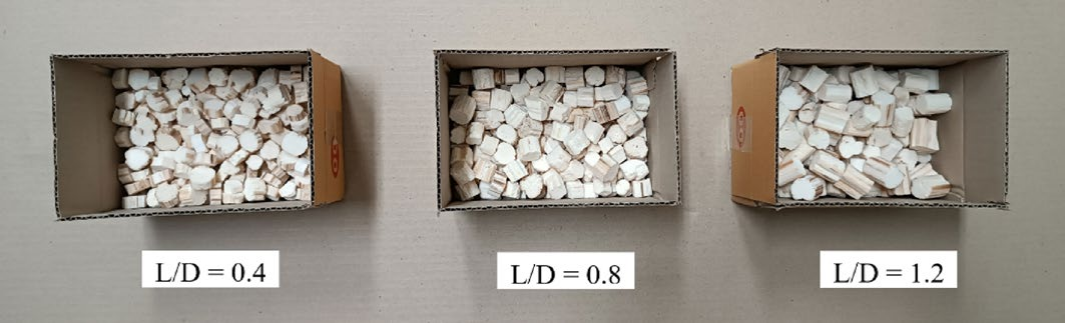
Appearance of the DCS^-R^P with a L/D ratio of 0.4, 0.8, and 1.2.

This study complied with the relevant institutional guideline. Fresh CS of waxy corn (Fig. 1a) were purchased and cut after harvest from a corn field in Doi Saket district, Chiang Mai province, Thailand. They were then cleaned with water and dried under sunlight for 3 days. The internodal CS^-R^ (Fig. 1b) was used in this work and was dried in a hot air oven at 105 °C for 7 h (the moisture content of the obtained DCS^-R^P was 2.4 ± 0.2%) and then kept in a desiccator at room temperature for at least 48 h prior to testing. A total of 45 DCS^-R^P (average diameter of 1.4 ± 0.1 cm) were then cut to L/D ratios of 0.4, 0.8, and 1.2 to form the respective DCS^-R^P (Fig. 2). The commercial EPS-based loose-fill packaging (W and S shapes), used for comparison.

### Cell diameter

The cell diameter of the DCS^-R^P was measured under a stereo microscope (Leica S8 APO, Germany) using the Leica application suite version 3.3 software. The measurement in both the radial and longitudinal directions of the cell was collected from 10 samples and the average values are reported.

### Water contact angle (WCA)

The WCA of the DCS^-R^P was determined using a Drop shape analyzer (DSA 30E, Krüss, Germany). Distilled water was dropped onto the surface of the cross-sectional DCS^-R^P and the WCA was measured from 12 samples using the Sessile drop method.

### Chemical analysis and Fourier transform infrared (FTIR) spectroscopy

The DCS^-R^P were prepared as extractive free samples by sequential extraction with (i) ethanol–benzene mixture, (ii) ethanol, and (iii) distilled water as reported (TAPPI T264 cm-97^28^). The lignin and alpha-cellulose were quantified according to TAPPI T222 om-06^29^ and TAPPI T203 cm-99^30^. Holo-cellulose was determined by the chloride method^31^. The analysis result was determined in triplicate. Moreover, the DCS^-R^P were ground in a mortar and mixed with potassium bromide to form a pellet. The infrared spectrum was collected using a FTIR spectrometer (47,000 type A, Jasco, Japan) at a resolution of 4 cm^−1^ and using 64 scans from 5000 to 350 cm^−1^.

### Water absorption and moisture absorption

The DCS^-R^P with L/D ratios of 0.4, 0.8, and 1.2 were submersed in distillated water (26 °C) for 0.5, 3, 5, 7, 9, 11, and 15 min. The water weight gain was calculated and recorded as the percentage of water absorption. The measurements were taken in triplicate. In addition, the DCS^-R^P were placed in various saturated salt solution desiccators—LiCl (11% relative humidity [RH]), MgCl_2_ (32% RH), Mg(NO_3_)_2_ (52% RH), NaCl (75% RH), KNO_3_ (93% RH), and distilled water (100% RH) and their moisture absorption was measured after 1, 2, 3, 4, 5, 6, 7, and 9 days and recorded from five samples.

### Thermal stability and derivative thermogravimetric (DTG) analyses

The thermal stability of the DCS^-R^P was investigated using thermogravimetric analysis (TGA; Mettler Toledo, TGA/DSC3+HT, Switzerland). Each sample of DCS^-R^P (approximately 6–8 mg) was placed in an alumina crucible and the analysis was performed from 25 to 1000 °C in a nitrogen or an oxygen atmosphere at a flow rate of 20 mL/min and a heating rate of 10 °C/min. Evaluation of the thermal stability was performed in triplicate. The DTG was also evaluated in a nitrogen or an oxygen atmosphere.

### X-ray diffraction (XRD)

The DCS^-R^P were ground using an analytical mill (IKA^®^ A11 basic, Germany) and passed through a 250-μm sieve. The diffraction pattern was recorded using a Rigaku X-ray diffractometer (Smartlab, Japan) with Cu Kα radiation and operated at 40 kV and 30 mA. The scanning was performed over a 2*θ* range of 5°–60° at a scan rate of 10°/min and a step size of 0.01°. The crystallinity index (CrI) was measured by the peak height method and calculated from Eq. (1)^32^:

1 $${\text{CrI }}\left( \% \right) = \frac{{\left( {I_{002} - I_{am} } \right)}}{{I_{002} }} \times 100$$

where *I*_002_ is the maximum intensity of the 002 lattice diffraction and *I*_*am*_ is the intensity of diffraction in the same unit at a 2*θ* of 18°. The CrI was calculated from two samples.

### Density, bulk density, and packing efficiency

The density of the DCS^-R^P with a L/D of 0.4, 0.8, and 1.2 (10 samples) was calculated from the weight and volume in g/cm^3^. Furthermore, the bulk density was measured using a box with an internal dimension of 7.2 cm × 7.2 cm × 7.7 cm (width × length × height). The DCS^-R^P with a given L/D ratio (0.4, 0.8, and 12) were filled in the box by free-fall dropping, and measured 10 times. The bulk density was calculated from the weight of the DCS^-R^P and the volume of the box in g/cm^3^. In addition, the packing efficiency was calculated from the bulk density divided by the density of DCS^-R^P.

### Compression test

The compression test was performed using a Texture analyzer (TA.XT.Plus, United Kingdom) with a load cell of 50 kg. The DCS^-R^P of the given L/D ratio (0.4, 0.8, and 1.2; 10 samples for each ratio) were compressed in both the perpendicular- and parallel-to-length dimensions using a 50-mm-diameter cylindrical probe. The compressive force was measured to a 50% strain at a test speed of 0.2 mm/s. In addition, the compressive failure pattern of the DCS^-R^P was investigated. The collapsing (%) was calculated from Eq. (2):

2 $${\text{Collapsing}} \left( \% \right) = \frac{{\left( {{\text{Initial}} {\text{ height}} {\text{ of}} {\text{ DCS}}^{{\text{ - R}}} {\text{P}} - {\text{Height of DCS}}^{{\text{ - R}}} {\text{P after compression}}} \right)}}{{{\text{Initial height of DCS}}^{{\text{ - R}}} {\text{P}} }} \times 100$$

**Figure 3:**
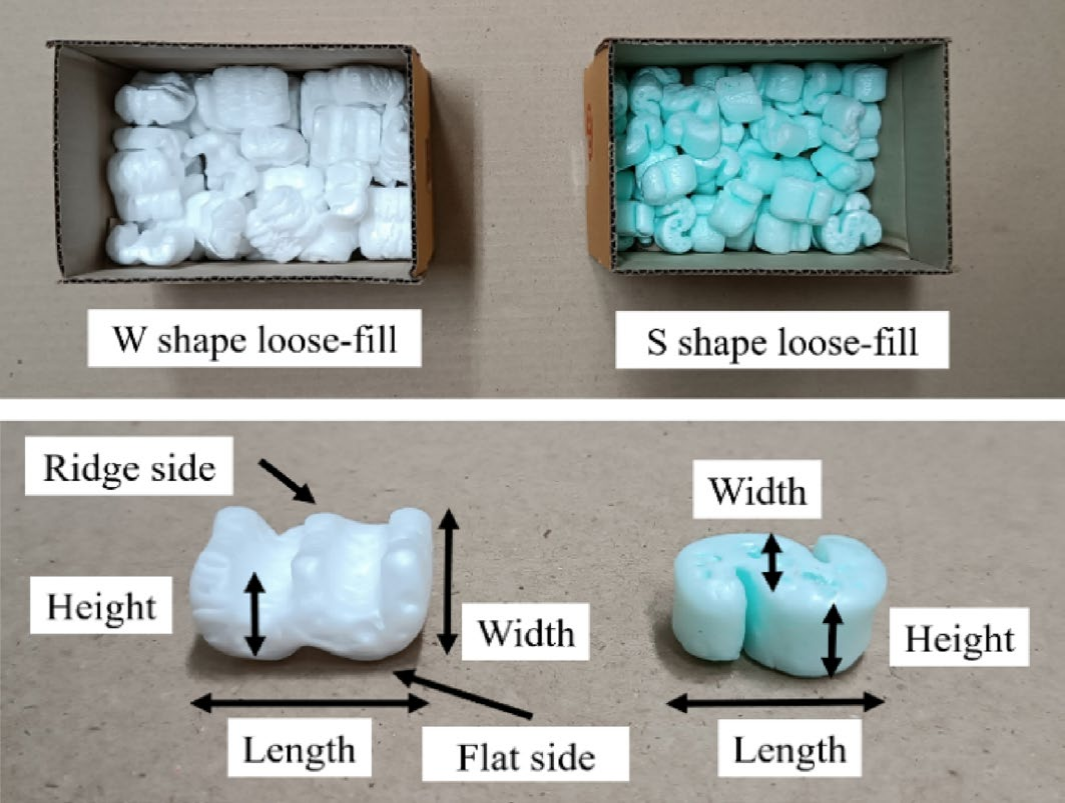
Commercial loose-fill with W and S shapes.

The compressive properties of the commercial loose-fill with W and S shapes (Fig. 3) were also examined for comparison, where those compressed in a parallel-to-height of loose-fill had an average width × length × height of the W-shaped loose-fill (10 samples) of 2.9 ± 0.1 cm × 3.2 ± 0.1 cm × 1.7 ± 0.1 cm while the S-shaped ones (10 samples) were 1.0 ± 0.1 cm × 2.7 ± 0.1 cm × 1.7 ± 0.1 cm. Furthermore, the W-shaped loose-fill was contacted with the cylindrical probe in both the ridge and flat side in the compression test. However, the commercial loose-fill was not compressed in the perpendicular-to-height direction in this study because the loose-fill could not stand alone by itself.

Moreover, the DCS^-R^P (9 g) of the given L/D ratio (0.4, 0.8, and 1.2) were filled in a box of an internal dimension of 7.0 cm × 7.0 cm × 8.8 cm (width × length × height) by free-fall dropping and then compressed using a rectangular plate of 6.9 cm × 6.9 cm × 0.4 cm (width x length x height) at a test speed of 0.2 mm/s and the maximum compressive force of the bulk DCS^-R^P to 50% strain was recorded in triplicate. Commercial loose-fill was also filled in the box to a similar volume as the filled DCS^-R^P. In addition, the W-shaped loose-fill was randomly packed in both the ridge and flat side.

## Results and discussion

### Appearances

**Figure 4:**
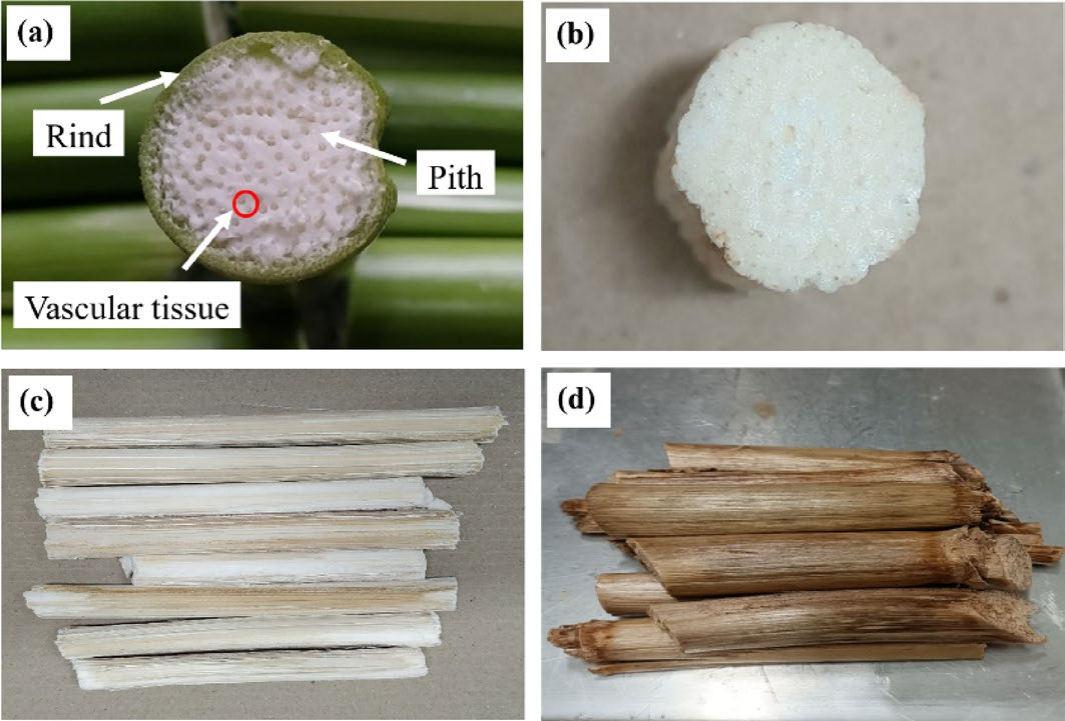
Cross-sectional photographs of (**a**) fresh CS^+R^ and (**b**) DCS^-R^, and the (**c**, **d**) outer longitudinal surface photograph of the (**c**) DCS^-R^ and (**d**) DCS^+R^.

The cross-section appearance of the fresh CS^+R^ and dried CS^-R^ (DCS^-R^) is presented in Fig. 4. For the fresh CS^+R^ (Fig. 4a), the outer part, as dermal tissue or rind, was hard and strong. For the inner part, the ground tissue (pith) was white and soft, while the vascular tissue was yellowish and exhibited a tube structure. After drying the fresh CS^+R^, the pith was softer, while the tube-structured vascular tissue was no longer soft. The pith of the DCS^-R^ was white when observed in cross-section (Fig. 4b) and its outer surface in the longitudinal direction exhibited various colors in one piece: white, yellowish, brownish, and brown (Fig. 4c). In addition, the vascular tissue of the DCS^-R^ was brownish in both the cross-section and outer longitudinal surface (Fig. 4b,c). After drying the fresh CS^+R^, the dried CS^+R^ (DCS^+R^) with a now brown rind (Fig. 4d) was very hard to be pressed in both the cross-sectional and longitudinal direction due to the hard and strong properties of the rind. Hence, the DCS^-R^ was softer after drying. Moreover, the toughness of the dried rind made it difficult to cut it to form the DCS^+R^P, whereas the DCS^-R^ could be cut easily to produce the desired L/D ratio sized DCS^-R^P and so was selected as a raw material for a bio loose-fill packaging in this work.

### Cell diameter

**Figure 5:**
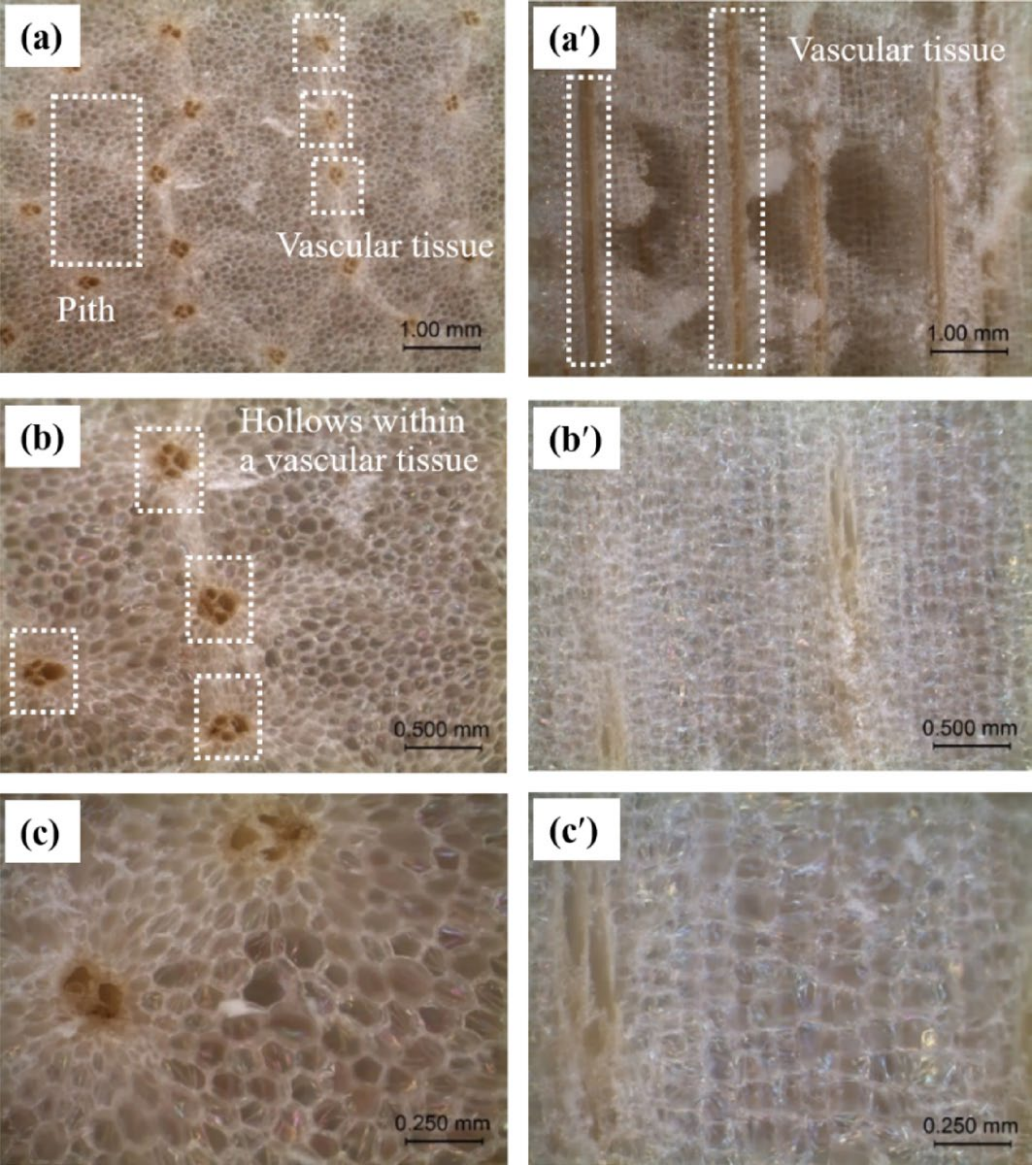
Representative optical micrographs of the DCS^-R^P showing the (**a**–**c**) radial and (**a**′–**c**′) longitudinal cross-section at (**a**, **a**′) 20 × , (**b**, **b**′) 40 × , and (**c**, **c**′) 80 × magnification.

Optical micrographs of the radial and longitudinal cross-sections of the DCS^-R^P are presented in Fig. 5. The cell diameter of the DCS^-R^P is reported for both the pith and vascular tissue. The average cell diameter of the pith and vascular tissue was 0.09 ± 0.02 mm and 0.24 ± 0.06 mm, respectively. A honeycomb structure with a hexagonal like shape was found in the DCS^-R^ pith in the radial cross-section. This was consistent with a previous scanning electron microscopy (SEM) based analysis of the CS^+R^ structure after harvesting^4^. Furthermore, a long tube (Fig. 5a′) with three or four hollows (Fig. 5b,c) within a tube was observed in the vascular tissue. After drying, air occupied inside the pith and vascular tissue and the DCS^-R^ changed to a softer porous structure. Sun et al.^6^ reported that the uniform pores were distributed inside the tube wall of the vascular tissue of the DCS^-R^, as observed in the SEM images. Therefore, the enormous porous structure of both the pith and vascular tissue in the DCS^-R^P could form a lightweight material. This lightweight characteristic is one of the required properties for a low cost loose-fill material for packaging prior to transportation.

### The WCA

**Figure 6:**
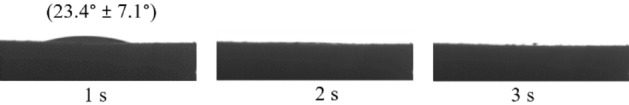
Water drop shape on the surface of DCS^-R^P. *The WCA value is given in parentheses.

The water drop shape on the DCS^-R^P is shown in Fig. 6, but was visible for only the first second as after that (at 2nd and 3rd sec), the water was absorbed inside the DCS^-R^P surface. The WCA was 23.4° ± 7.1°, which is consistent with the previously reported super hydrophilic nature (structure) of the DCS^-R^P, where the water drop was present for 0.8 s and absorbed within 3 s to cover 20% of the surface of the DCS^-R^P (pieces of 20 mm diameter and 10 mm height)^6^. The low WCA and fast water absorption of the DCS^-R^P surface were due to the porous structure of the pith and tube of the vascular tissue (Fig. 5). For application, the DCS^-R^P as a bio loose-fill should be kept away from water before use and applied for dry product packaging since the surface of the DCS^-R^P absorbed water in a short time.

### Chemical analysis and FTIR spectroscopy

**Figure 7:**
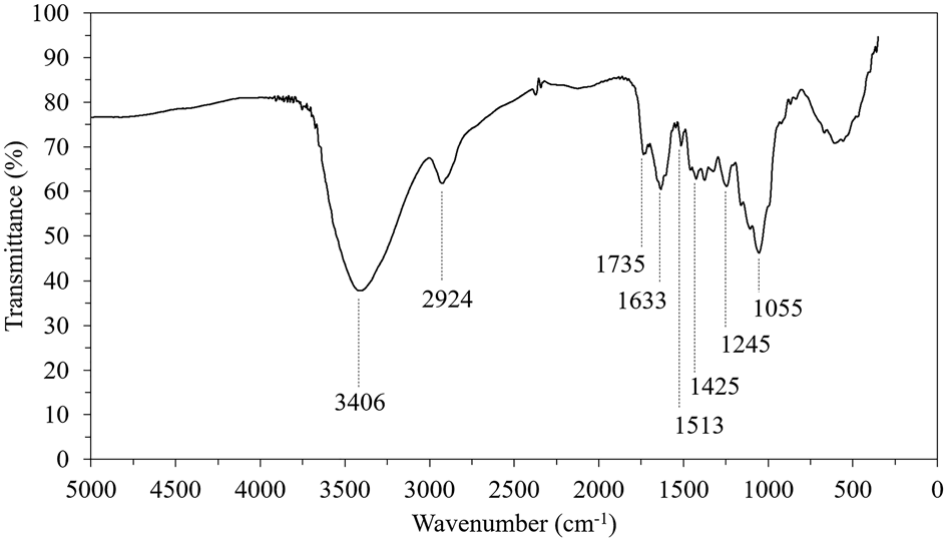
Representative FTIR spectrum of DCS^-R^P.

The DCS^-R^P was found to contain lignin at 27.6 ± 6.0%, holo-cellulose at 70.2 ± 0.8%, and alpha-cellulose at 50.1 ± 0.3% (all on a dry basis based on dry holo-cellulose). Figure 7 shows a representative FTIR spectrum of the DCS^-R^P. The O–H stretching of the hydrogen bond at 3406 cm^−1^ was attributed to the hydroxyl group of cellulose and hemicellulose^33^, while the absorption peak at 2924 cm^−1^ was attributed to the C-H stretching of cellulose^34^. The ester group was reflected in the peak at 1735 cm^−1^^35^. The absorption band at 1245 cm^−1^ was related to the β-O-4 ether band^34^, while the C–O stretching of cellulose was reflected in the peak at 1054–1055 cm^−1^^34,36^. The bands at 1633, 1513, and 1425 cm^−1^ were the aromatic ring stretching in lignin^33,36^. From these composition results, the DCS^-R^P would be predicted to be biodegradable, which is one method to manage the waste at the end of the lifespan of any DCS^-R^P-based bio loose-fill packaging. This would improve (lessen) the environmental impact from previous petroleum-based loose-fill packagings.

### Water absorption and moisture absorption

The water absorption of the DCS^-R^P with different L/D ratios (0.4, 0.8, and 12) during 15 min was higher than 100% of its dry weight, as presented in Table 1. The porous structure and hollow tubes in the DCS^-R^P (Fig. 5) could absorb and hold water after water immersion. In addition, the moisture absorption by the DCS^-R^P increased at increasing %RH values for all three L/D ratios of DCS^-R^P (Table 2), while fungal growth was observed at higher RH values—after 6 days at 93% RH and 3 days at 100% RH. The numerous pores in the DCS^-R^P could hold moisture in their porous structure via hydrogen bonding with the hydroxyl groups, the presence of which was confirmed in the FTIR spectrum (Fig. 7), which would attract moisture and so play a role in moisture absorption. As the DCS^-R^P could absorb and hold water after water immersion or exposure to a high RH atmosphere, the DCS^-R^P-based bio loose-fill packaging should be kept far from water and in a dry (low RH) condition before use. Otherwise, the absorbed moisture will degrade and reduce the usage time of the DCS^-R^P.

**Table 1:** Mean water absorption of DCS^-R^P with a L/D ratio of 0.4, 0.8, and 1.2.

L/D	Water absorption (%)						
	Time (min)						
	0.5	3	5	7	9	11	15
0.4	254.9	315.3	369.1	396.0	419.9	448.2	476.3
0.8	232.9	338.3	365.4	403.6	423.3	444.2	462.2
1.2	86.9	141.2	166.7	187.2	208.7	223.2	236.4

The SD of each mean water absorption (%) was in the range of 6.7–60.7% (see supplementary Table S1).

**Table 2:** Mean moisture absorption of DCS^-R^P with a L/D ratio of 0.4, 0.8, and 1.2.

RH (%)	L/D	Moisture absorption (%)							
		Time (day)							
		1	2	3	4	5	6	7	9
32	0.4	–	–	–	–	–	–	–	1.3
	0.8	4.0	4.6	4.1	4.3	4.4	4.2	4.2	4.2
	1.2	2.6	3.1	2.6	3.1	3.1	2.7	2.6	2.6
52	0.4	7.1	7.5	7.6	7.0	7.4	7.6	7.5	7.6
	0.8	6.7	7.4	7.1	7.8	7.0	7.3	6.7	6.9
	1.2	6.6	7.0	6.5	7.1	6.8	6.9	6.6	7.2
75	0.4	19.5	20.1	20.4	18.8	20.4	20.7	20.6	21.2
	0.8	15.7	16.2	16.9	16.3	16.6	17.1	15.9	17.0
	1.2	15.2	16.7	16.0	16.7	17.0	16.9	16.2	17.3
93	0.4	40.9	50.7	53.7	52.6	55.2	51.9	Fungi	Fungi
	0.8	36.5	46.8	49.1	49.7	49.1	50.7	Fungi	Fungi
	1.2	40.7	38.7	41.3	44.4	43.0	43.1	Fungi	Fungi
100	0.4	100.3	127.7	151.2	Fungi	Fungi	Fungi	Fungi	Fungi
	0.8	82.4	113.4	136.6	Fungi	Fungi	Fungi	Fungi	Fungi
	1.2	76.5	107.8	124.5	Fungi	Fungi	Fungi	Fungi	Fungi

Note, the weight of all samples did not change at an 11% RH so the data is omitted. The SD of each mean moisture absorption (%) was in the range of 0.4–23.4% (see supplementary Table S2). Fungi = reading was not possible due to fungal growth.

### Thermal stability and properties

**Figure 8:**
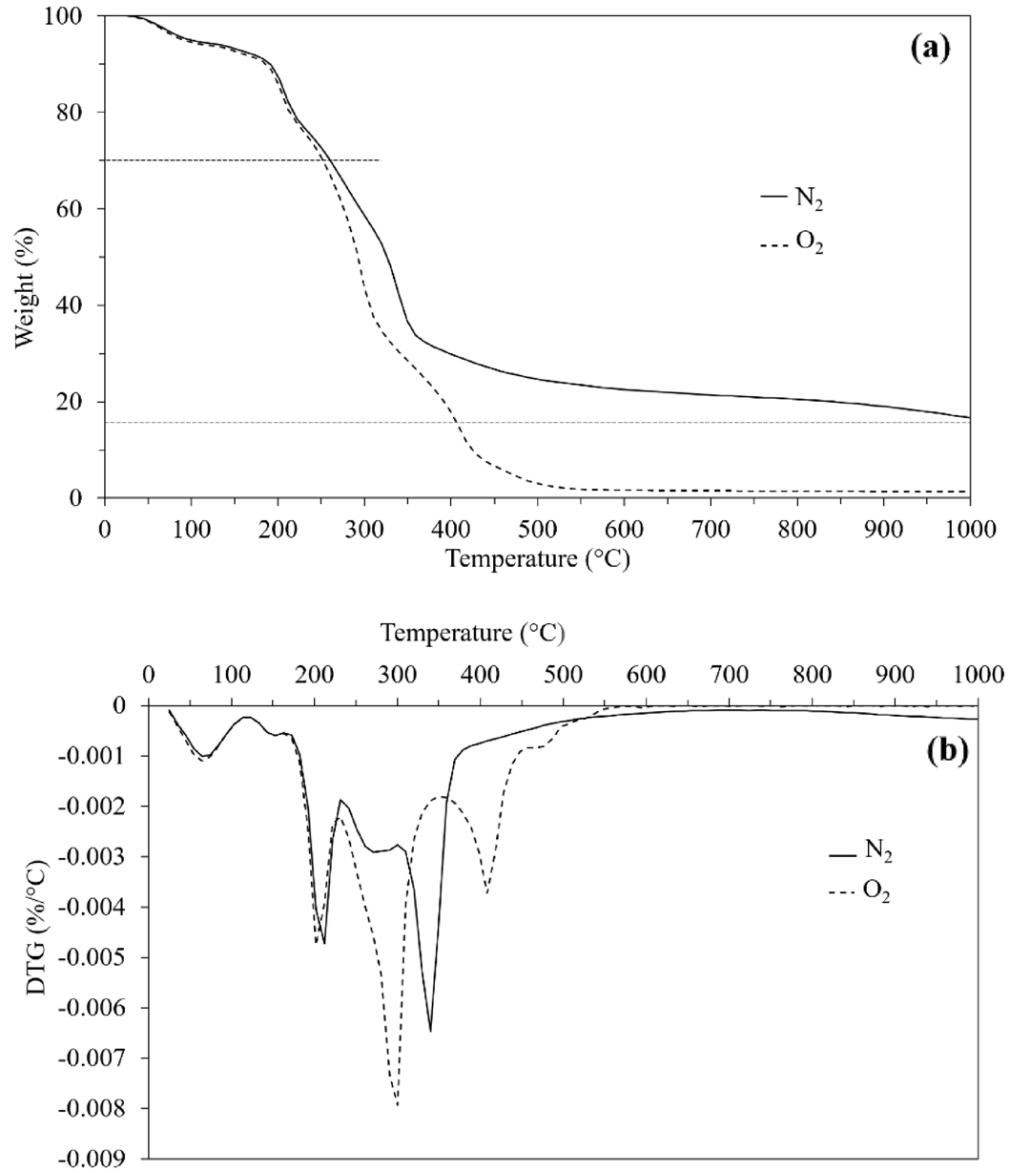
Representative (**a**) thermogravimetric curves of DCS^-R^P in a N_2_ or an O_2_ atmosphere and (**b**) DTG curves of the DCS^-R^P in a N_2_ or an O_2_ atmosphere.

The pyrolysis in a nitrogen atmosphere and combustion in an oxygen atmosphere of the DCS^-R^P were evaluated using TGA. The DCS^-R^P exhibited a multi-step thermal decomposition when undergoing either pyrolysis or combustion (Fig. 8a). The initial decomposition stage gave a 30% weight loss and revealed a similar decomposition step and decomposition temperature in both the pyrolysis and combustion. After a 30–85% weight loss, the decomposition temperature of the DCS^-R^P in a nitrogen atmosphere was higher than that in an oxygen atmosphere. Therefore, the pyrolysis of DCS^-R^P requires a higher temperature than its combustion, which implies that the thermal stability of the DCS^-R^P in pyrolysis was higher than that in combustion. In addition, the residual weight after pyrolysis was higher than that after combustion at 1000 °C (Table 3).

**Table 3:** Mean residue weight and DTG peak temperature of DCS^-R^P during thermal decomposition.

Gas	Weight (%) at 1000 °C	1st DTG peak (°C)	2nd DTG peak (°C)	3rd DTG peak (°C)	4th DTG peak (°C)
N_2_	14.9	66.5	208.4	339.6	–
O_2_	1.2	66.7	205.4	297.3	410.6

The SD of each mean residue weight (%) at 1000 °C and DTG peak temperature were in the range of 0.3–1.9 and 0.2–3.3, respectively, (see supplementary Table S3).

The DTG curves of pyrolysis and the DTG peak temperature are illustrated in Fig. 8b and Table 3, respectively. The pyrolysis of the DCS^-R^P consisted of three stages. Stage one, at 25–170 °C, represents the drying stage where water was released from the DCS^-R^P. Stage two, at a temperature range of 170–500 °C, was the pyrolytic decomposition stage or main pyrolysis, where cellulose and hemicellulose were decomposed, as seen in the DTG curve peaks^7,37^. The peak at 339.6 °C (3rd DTG peak) could be the decomposition of linear chain cellulose, which degraded at a higher temperature than the side and branch chains of hemicellulose^38^. Moreover, the sharp peak at about 337 °C, corresponding to cellulose pyrolysis, was previously also observed in the DTG curve of the acid-detergent prepared fibers of corn stover^38^. From Fig. 8b, the peak at 208.4 °C (2nd DTG) corresponded to the decomposition of hemicellulose in the DCS^-R^P. Stage three, at a temperature of more than 500 °C, was the carbonization stage, and was the slow decomposition of solid residue^7,37^. At 1000 °C, a black solid residue was obtained. Although, lignin could be decomposed in both the main pyrolysis and carbonization stages^37,39^, the lignin decomposition peak of the DCS^-R^P was not observed, which is consistent with a previous report on DCS^+R^^37^.

The DTG curves of combustion and the DTG peak temperature are illustrated in Fig. 8b and Table 3, respectively. As with pyrolysis, the combustion of the DCS^-R^P also consisted of three stages. Stage one, at a temperature range of 25–170 °C, was the drying stage, where water was removed from the DCS^-R^P. Stage two, at a temperature range of 170–550 °C, was the combustion decomposition stage (main combustion). Three decomposition peaks were revealed at this stage. The first two peaks at 205.4 °C and 297.3 °C corresponded to the decomposition of hemicellulose and cellulose, respectively. Close temperature peak values for hemicellulose decomposition (2^nd^ DTG peak) were obtained in both the main combustion and pyrolysis. However, the peak temperature of cellulose decomposition (3rd DTG peak) in the main combustion shifted to a lower temperature than that in the main pyrolysis. Furthermore, the peak at 410.6 °C (4th DTG peak) could be the decomposition of lignin. Theng et al.^12^ reported the decomposition temperature of kraft lignin at about 450 °C in an air atmosphere. Therefore, various lignin decomposition temperatures in the presence of oxygen have been obtained, depending on the plant source.

The third decomposition stage, at a temperature higher than 500 °C, was the slow decomposition stage. At 1000 °C, the DCS^-R^P approached a nearly complete combustion state with only a tiny amount of white solid residue being obtained. In addition, a solid black residue was also observed at 800 °C and became white after combustion, which indicated the absence of unburnt carbon^40^, whereas a black solid residue of unburnt carbon was still obtained in the pyrolysis even at 1000 °C.

Pyrolysis and direct combustion of CS are well-known methods to convert biomass to energy^8,40^, and pyrolysis can produce value-added products from CS^7^. In addition, calcination of CS produced a CS ash that could be used as a pozzolan for eco-friendly cement/concrete^40^. Therefore, the TGA of the pyrolysis and combustion of DCS^-R^P could give the decomposition behavior for further management of waste DCS^-R^P as bio loose-fill materials after their end use, such as in packaging waste management. However, different solid residue contents were obtained depending on the gas atmosphere and temperature.

### Composition of the DCS^-R^P: XRD analysis

**Figure 9:**
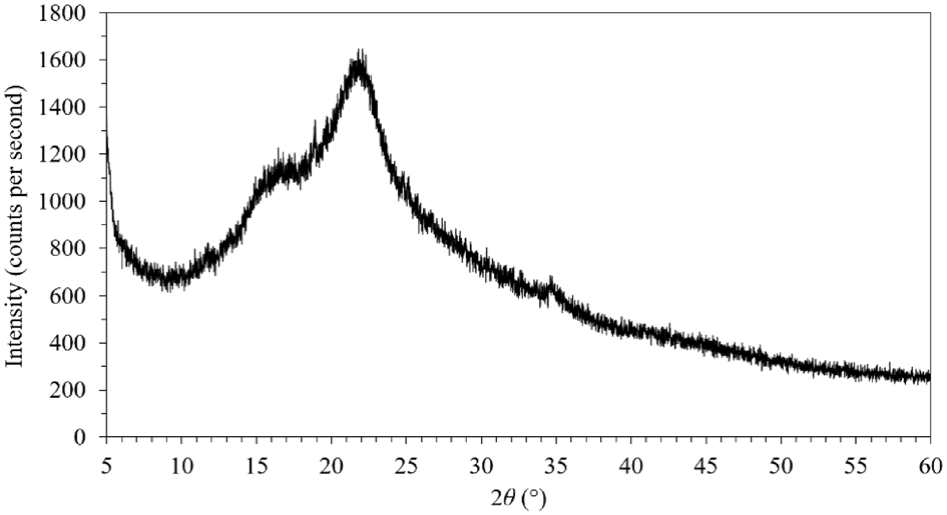
Representative XRD pattern of DCS^-R^P.

The XRD pattern of the DCS^-R^P is shown in Fig. 9. The maximum intensity at a 2*θ* of 22°–23° corresponds to the 002 lattice diffraction, while the intensity at a 2*θ* of 18° indicated the pattern of amorphous cellulose^32^. The CrI of the DCS^-R^P was 30.5%, which was lower than those reported before for CS^+R^ at 33.2%^14^, 35.6%^33^, and 51.8%^36^. It can be seen the variation in the CrI values of CS^+R^ depended on the CS type^14,33,36^. As the crystallinity affects the strength of the material, the DCS^-R^ was softer than the DCS^+R^. Consequently, the DCS^-R^ was easier to cut than the DCS^+R^ to form tube-shaped bio-loose-fill packaging pieces.

### Density, bulk density, and packing efficiency

The density of DCS^-R^P with a L/D ratio of 0.4, 0.8, and 1.2 ranged between 0.108 and 0.086 g/cm^3^. However, the bulk density of DCS^-R^P with a L/D ratio of 0.4 and 0.8 was slightly higher than that with a L/D ratio of 1.2, which was due to the higher size and longer cylindrical shape of the DCS^-R^P with a L/D ratio of 1.2. The packing efficiency values of the different DCS^-R^P were all lower than 1 (Table 4), where a packing efficiency of 1 means no void volume between adjacent DCS^-R^P on packing^23^. However, the packing efficiency values for the DCS^-R^P with different L/D ratios were only slightly different (0.38–0.44), and so the different L/D ratios did not markedly affect the voids in packing. The low density DCS^-R^P resulted in a lightweight bio loose-fill packaging, a good characteristic as the transportation cost depends on the weight inside the packages.

**Table 4:** Mean density, bulk density, and packing efficiency of DCS^-R^P with a L/D ratio of 0.4, 0.8, and 1.2.

L/D	Density (g/cm^3^)	Bulk density (g/cm^3^)	Packing efficiency
0.4	0.108	0.041	0.38
0.8	0.092	0.041	0.44
1.2	0.086	0.036	0.42

The SD of each mean density (g/cm^3^), bulk density (g/cm^3^), and packing efficiency ranged from 0.013–0.026, 0.001–0.002, and 0.01–0.02, respectively, (see supplementary Table S4).

### Compression test

The maximum compressive force in the parallel- and perpendicular-to-length orientation of the DCS^-R^P with different L/D ratios are presented in Table 5. The compression of DCS^-R^P in the parallel-to-length orientation was more difficult than that of the perpendicular-to-length orientation. As the long tube vascular tissue aligned in the vertical direction (Fig. 5a′), the compressive force was parallel to the length orientation of DCS^-R^P. In contrast, the long tube vascular tissue aligned in a horizontal direction, where the compression force was in the perpendicular-to-length orientation of the DCS^-R^P. This implied that when the long tube vascular tissue was aligned in a vertical direction it could resist the compression force better than when aligned in a horizontal direction. Moreover, the resistance to compressive force of the pith in the radial cross-section (Fig. 5c) was higher than in the longitudinal cross-section (Fig. 5c′). The DCS^-R^P with a L/D ratio of 0.8 and 1.2 were difficult to compress in the parallel-to-length orientation compared to those with a L/D ratio of 0.4. Thus, the small-sized DCS^-R^P (at a L/D ratio of 0.4) could resist the compression force less than the large-sized DCS^-R^P (at a L/D ratio of 0.8 and 1.2).

**Table 5:** Compressive force of DCS^-R^P with L/D ratios of 0.4, 0.8, and 1.2 (*at 50% strain and ** at 30% strain).

L/D	Maximum compressive force (kg)			Collapsing (%)
	Parallel to length	Perpendicular to length	Bulk DCS^-R^P	Parallel to length
0.4	9.16 ± 1.06	1.10 ± 0.49	46.03 ± 5.45*	19 ± 7
0.8	29.04 ± 7.27	1.85 ± 0.22	18.51 ± 3.98**	38 ± 5
1.2	29.05 ± 3.73	3.05 ± 0.32	19.30 ± 9.38**	42 ± 3

Data are shown as the mean ± 1SD.

**Figure 10:**
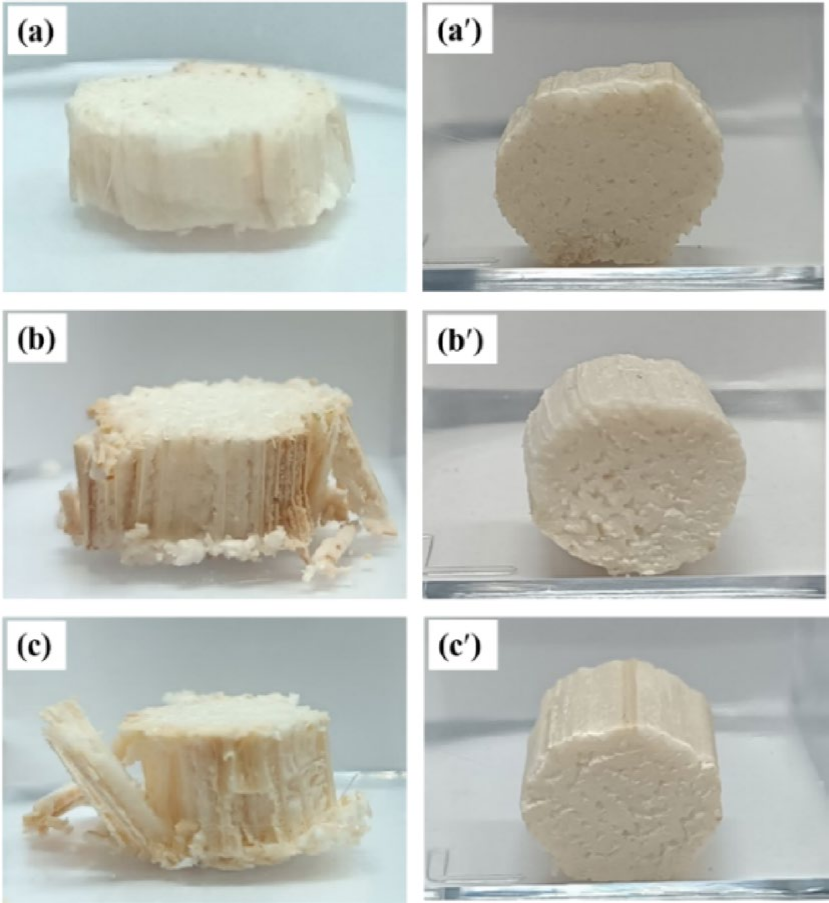
Compressive failure pattern in the (**a**–**c**) parallel- and (**a**′–**c**′) perpendicular-to-length orientation of the DCS^-R^P with a L/D ratio of: (**a**, **a**′) 0.4, (**b**, **b**′) 0.8, and (**c**, **c**′) 1.2.

The compressive failure pattern of each DCS^-R^P with a different L/D ratio is presented in Fig. 10. After unloading, the DCS^-R^P with a L/D ratio of 0.4 revealed a slight collapse without any splits, whereas those with L/D ratios of 0.8 and 1.2 exhibited a greater degree of collapse and split failure. Since the DCS^-R^P with a L/D ratio of 0.4 used a shorter compression time to a 50% strain than those with a L/D ratio of 0.8 and 1.2, the small-sized DCS^-R^P (a L/D ratio of 0.4) only had time for initial damage, while the large-sized DCS^-R^P (a L/D ratio of 0.8 and 1.2) had enough time for damage propagation. However, all the DCS^-R^P did not recover to their original height after unloading. This indicated that the long tube vascular tissue aligned in the vertical direction and the pith in the radial cross-section did not exhibit resilient properties (the compressive force was in a parallel-to-length orientation of the DCS^-R^P). From Table 5, the collapse of DCS^-R^P was presented after deformation to a 50% strain, and the % collapse in the parallel to the length direction of the DCS^-R^P with a L/D ratio of 0.4 (19%) was significantly lower than those with a L/D ratio of 0.8 and 1.2 (38% and 42%, respectively).

The maximum compressive force in the perpendicular to length orientation of the DCS^-R^P increased with an increasing L/D ratio (Table 5). At all three L/D ratios, the maximum compressive force in the perpendicular-to-length direction was lower than that in the parallel-to-length direction. After unloading, the compressive failure pattern of the compressive force when perpendicular to the length of the DCS^-R^P is shown in Fig. 10. For all L/D ratios, the DCS^-R^P exhibited no split failure pattern but was slightly flattened from its original shape. Therefore, the DCS^-R^P was softer in the direction perpendicular to the compressive force compared to that when parallel to the compressive force. In addition, the slightly flat pattern of the DCS^-R^P from its original shape revealed that the DCS^-R^P could not totally recover to its original shape after loading in a length direction perpendicular to the compressive force. Whereas, Kovács and Kerényi^19^ found that a straight vertical break appeared in the core at the initial failure pattern of CS^+R^ when a compressive force was applied perpendicular to its length. After that the failure propagated to the rind and split it into two separate parts, and then horizontal breaks of the rind and a flattened shape were revealed. Therefore, different failure patterns could be observed between CS^+R^ (ref 19) and DCS^-R^ (this study).

**Figure 11:**
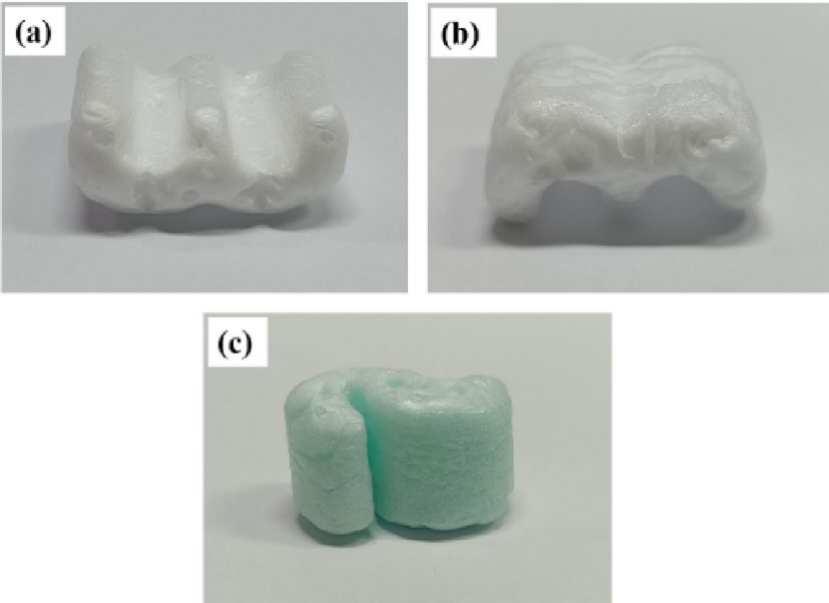
Compressive failure pattern of the commercial ESP-based (**a**, **b**) W-shaped loose-fill with (**a**) ridge side contact and (**b**) flat side contact with cylindrical probe, and (**c**) S-shaped loose-fill.

For comparison of the DCS^-R^P and commercial loose-fill, the maximum compressive force of the commercial loose-fill is presented in Table 6. The DCS^-R^P (Table 5) exhibited a higher compressive force than the commercial loose-fill for both W- and S-shaped loose-fills. From the failure pattern after unloading (Fig. 11), the commercial W- and S-shaped loose-fill did not show the fracture similar to the DCS^-R^P, but rather they could recover to their original size (due to polymeric foam behavior) after unloading, while the DCS^-R^P samples remained collapsed. Therefore, the commercial loose-fill packaging could be reused several times in contrast to the single-use DCS^-R^P.

**Table 6:** Compressive force of a commercial EPS-based loose-fill.

Commercial EPS-based loose-fill	Maximum compressive force (kg)	
	Parallel to height	Bulk commercial loose-fill
W-shaped (ridge side contact with cylindrical probe)	2.80 ± 0.38	8.46 ± 0.31 (random packed in both ridge and flat sides)
W-shaped (flat side contact with cylindrical probe)	2.71 ± 0.48	
S-shaped	2.00 ± 0.44	10.67 ± 0.09

Data are shown as the mean ± 1SD.

**Figure 12:**
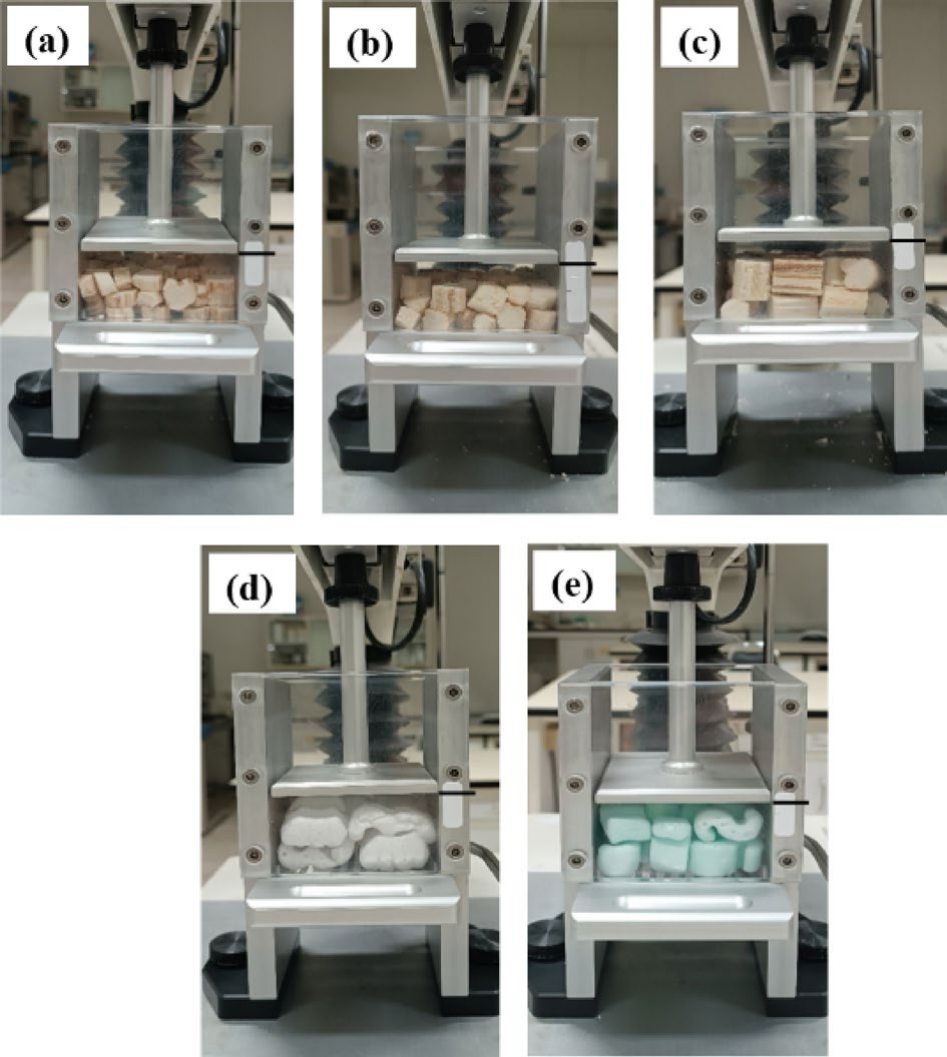
Compressive failure pattern of the (**a**–**c**) bulk DCS^-R^P with L/D ratios of: (**a**) 0.4, (**b**) 0.8, and (**c**) 1.2, and the bulk (**d**) W-shaped and (**e**) S-shaped loose-fills (black line is original volume).

In the bulk compression test, the DCS^-R^P with a L/D of 0.8 and 1.2 could not be compressed to 50% strain due to the overload cell limit of the equipment. Therefore, the maximum compressive force at a 30% strain of DCS^-R^P with a L/D ratio of 0.8 and 1.2 was determined and was found to be broadly similar (Table 5). The compressive force in the perpendicular-to-length orientation of the DCS^-R^P with a L/D ratio of 1.2 was higher than that with a L/D ratio of 0.8, which may reflect that the random distribution of parallel and perpendicular orientations in each packing affected the compressive resistance of the bulk DCS^-R^P. The compressive failure pattern of bulk DCS^-R^P with different L/D ratios (Fig. 12) revealed a flattening pattern (volume) compared with the original volume of the bulk DCS^-R^P after unloading. In addition, each sample of DCS^-R^P exhibited no split failure due to the distribution of the compressive force on each DCS^-R^P in the random packing. Compared with the commercial ESP-based W- and S-shaped loose-fill, the compressive force of the bulk DCS^-R^P (Table 5) was higher for both the W- and S-shaped loose-fills (Table 6). Moreover, the compressive failure pattern of the bulk commercial W- and S-shaped loose-fill was similar to that for the bulk DCS^-R^P as a flattening pattern (volume) compared with the original volume (Fig. 12). In this case, the DCS^-R^P bio loose-fill packaging had the higher compressive resistance but recovered to its original size less than the commercial loose-fill packaging.

## Conclusion

A DCS^-R^P -based bio loose-fill packaging could be suitable as a lightweight void filling product. The porous structure of DCS^-R^P led to its lightweight nature and good properties as a loose-fill material. The bulk density of DCS^-R^P with a L/D ratio of 1.2 was slightly lower than those with a L/D ratio of 0.4 and 0.8. However, the L/D ratios did not markedly affect the packing efficiency. The compressive resistance of DCS^-R^P at all three tested L/D ratios in the parallel-to-length orientation exhibited a higher value than that in the perpendicular-to-length orientation. In addition, the L/D ratios also affected the ability of DCS^-R^P to resist the compression load. The compressive resistance of the bulk DCS^-R^P and DCS^-R^P with a L/D ratio of 0.8 and 1.2 were higher than those with a L/D ratio of 0.4. Thus, the DCS^-R^P with a L/D ratio of 0.8 and 1.2 had a good resistance to the compression load and a sufficient packing efficiency, giving them a high potential as a bio loose-fill packaging with appropriate waste management, such as pyrolysis, combustion, biodegradation. However, a flattening failure pattern (volume) was still retained after the end use of DCS^-R^P bio-loose fill in the form of the bulk DCS^-R^P.

### Supplementary Information

Supplementary Tables.

## Data Availability

The dataset generated and/or analyzed during the current study are available from the corresponding authors on reasonable request.
