# Supplementary material for: Morphological, physical, thermal, and mechanical properties with the aspect ratio effects of bio loose-fill packaging from corn stalk
Source: Sci Rep. 2023 Sep 12;13:15084. doi: 10.1038/s41598-023-41612-5 (PMC10497553; doi:10.1038/s41598-023-41612-5)
Supplement: Supplementary file 1 — Supplementary Tables. [file 41598_2023_41612_MOESM1_ESM.docx]

**Supplementary Information**

**Morphological, physical, thermal, and mechanical properties**

**with the aspect ratio effects of bio loose-fill packaging from corn stalk**

**Linda Thiraphattaraphun^1*^ & Pattarapan Prasassarakich^2,3,4*^**

^1^Division of Packaging Technology, Faculty of Agro-Industry, Chiang Mai University, Chiang Mai 50100, Thailand

^2^Department of Chemical Technology, Faculty of Science, Chulalongkorn University, Bangkok 10330, Thailand

^3^Center of Excellence on Petrochemical and Materials Technology, Chulalongkorn University, Bangkok 10330, Thailand

^4^Center of Excellence in Green Materials for Industrial Application, Chulalongkorn University, Bangkok 10330, Thailand

***Corresponding authors**

Linda Thiraphattaraphun, Email: [linda.t@cmu.ac.th](mailto:linda.t@cmu.ac.th)

Pattarapan Prasassarakich, Email: [ppattara@chula.ac.th](mailto:ppattara@chula.ac.th)

**Table S1.** Water absorption of DCS^-R^P with a L/D ratio of 0.4, 0.8, and 1.2. Data are shown as the mean ± 1 SD.

| L/D |  | Water absorption (%) | | | | | | |
| --- | --- | --- | --- | --- | --- | --- | --- | --- |
|  |  | Time (min) | | | | | | |
|  |  | 0.5 | 3 | 5 | 7 | 9 | 11 | 15 |
| 0.4 |  | 254.9 ± 22.3 | 315.3 ± 13.8 | 369.1 ± 22.3 | 396.0 ± 9.9 | 419.9 ± 25.9 | 448.2 ± 18.1 | 476.3 ± 15.0 |
| 0.8 |  | 232.9 ± 19.1 | 338.3 ± 14.6 | 365.4 ± 22.8 | 403.6 ± 27.1 | 423.3 ± 17.1 | 444.2 ± 18.5 | 462.2 ± 22.2 |
| 1.2 |  | 86.9 ± 6.7 | 141.2 ± 18.9 | 166.7 ± 28.6 | 187.2 ± 36.5 | 208.7 ± 51.2 | 223.2 ± 49.2 | 236.4 ± 60.7 |

**Table S2.** Moisture absorption of DCS^-R^P with a L/D ratio of 0.4, 0.8, and 1.2. Data are shown as the mean ± 1SD.

| RH (%) | L/D |  | Moisture absorption (%) | | | | | | | |
| --- | --- | --- | --- | --- | --- | --- | --- | --- | --- | --- |
|  |  |  | Time (day) | | | | | | | |
|  |  |  | 1 | 2 | 3 | 4 | 5 | 6 | 7 | 9 |
| 11 | 0.4 |  | - | - | - | - | - | - | - | - |
|  | 0.8 |  | - | - | - | - | - | - | - | - |
|  | 1.2 |  | - | - | - | - | - | - | - | - |
| 32 | 0.4 |  | - | - | - | - | - | - | - | 1.3  ± 0.8 |
|  | 0.8 |  | 4.0  ± 0.8 | 4.6  ± 0.8 | 4.1  ± 0.6 | 4.3  ± 1.0 | 4.4  ± 0.7 | 4.2  ± 0.7 | 4.2  ± 0.8 | 4.2  ± 0.8 |
|  | 1.2 |  | 2.6  ± 0.4 | 3.1  ± 0.4 | 2.6  ± 0.5 | 3.1  ± 0.6 | 3.1  ± 0.5 | 2.7  ± 0.6 | 2.6  ± 0.9 | 2.6  ± 0.6 |
| 52 | 0.4 |  | 7.1  ± 1.2 | 7.5  ± 1.4 | 7.6  ± 1.1 | 7.0  ± 1.4 | 7.4  ± 1.9 | 7.6  ± 2.5 | 7.5  ± 1.2 | 7.6  ± 2.0 |
|  | 0.8 |  | 6.7  ± 1.3 | 7.4  ± 1.2 | 7.1  ± 1.3 | 7.8  ± 1.6 | 7.0  ± 1.4 | 7.3  ± 1.1 | 6.7  ± 1.8 | 6.9  ± 1.9 |
|  | 1.2 |  | 6.6  ± 1.2 | 7.0  ± 1.1 | 6.5  ± 0.9 | 7.1  ± 1.0 | 6.8  ± 1.1 | 6.9  ± 1.3 | 6.6  ± 1.2 | 7.2  ± 1.4 |
| 75 | 0.4 |  | 19.5  ± 3.3 | 20.1  ± 3.5 | 20.4  ± 2.9 | 18.8  ± 3.3 | 20.4  ± 2.9 | 20.7  ± 2.5 | 20.6  ± 2.5 | 21.2  ± 3.2 |
|  | 0.8 |  | 15.7  ± 4.5 | 16.2  ± 5.0 | 16.9  ± 5.3 | 16.3  ± 5.3 | 16.6  ± 5.6 | 17.1  ± 5.8 | 15.9  ± 5.4 | 17.0  ± 5.7 |
|  | 1.2 |  | 15.2  ± 3.7 | 16.7  ± 3.7 | 16.0  ± 4.1 | 16.7  ± 4.0 | 17.0  ± 3.9 | 16.9  ± 3.8 | 16.2  ± 4.1 | 17.3  ± 4.0 |
| 93 | 0.4 |  | 40.9  ± 7.8 | 50.7  ± 11.7 | 53.7  ± 11.1 | 52.6  ± 11.9 | 55.2  ± 11.7 | 51.9  ± 10.9 | Fungi | Fungi |
|  | 0.8 |  | 36.5  ± 10.2 | 46.8  ± 15.1 | 49.1  ± 15.4 | 49.7  ± 16.5 | 49.1  ± 16.5 | 50.7  ± 15.5 | Fungi | Fungi |
|  | 1.2 |  | 40.7  ± 3.7 | 38.7  ± 7.9 | 41.3  ± 8.3 | 44.4  ± 9.4 | 43.0  ± 9.6 | 43.1  ± 9.7 | Fungi | Fungi |
| 100 | 0.4 |  | 100.3  ± 8.5 | 127.7  ± 7.7 | 151.2  ± 14.6 | Fungi | Fungi | Fungi | Fungi | Fungi |
|  | 0.8 |  | 82.4  ± 4.2 | 113.4  ± 12.5 | 136.6  ± 16.8 | Fungi | Fungi | Fungi | Fungi | Fungi |
|  | 1.2 |  | 76.5  ± 14.6 | 107.8  ± 17.2 | 124.5  ± 23.4 | Fungi | Fungi | Fungi | Fungi | Fungi |

**Table S3.** Residue weight and DTG peak temperature of DCS^-R^P during thermal decomposition. Data are shown as the mean ± 1SD.

| Gas | Weight (%) at 1,000 °C | 1^st^ DTG peak  (°C) | 2^nd^ DTG peak  (°C) | 3^rd^ DTG peak  (°C) | 4^th^ DTG peak  (°C) |
| --- | --- | --- | --- | --- | --- |
| N_2_ | 14.9 ± 1.9 | 66.5 ± 3.2 | 208.4 ± 0.2 | 339.6 ± 1.5 | - |
| O_2_ | 1.2 ± 0.3 | 66.7 ± 3.3 | 205.4 ± 0.2 | 297.3 ± 0.7 | 410.6 ± 0.8 |

**Table S4.** Density, bulk density, and packing efficiency of DCS^-R^P with a L/D ratio of 0.4, 0.8, and 1.2. Data are shown as the mean ± 1SD.

| L/D | Density (g/cm^3^) | Bulk density (g/cm^3^) | Packing efficiency |
| --- | --- | --- | --- |
| 0.4 | 0.108 ± 0.026 | 0.041 ± 0.002 | 0.38 ± 0.02 |
| 0.8 | 0.092 ± 0.021 | 0.041 ± 0.002 | 0.44 ± 0.02 |
| 1.2 | 0.086 ± 0.013 | 0.036 ± 0.001 | 0.42 ± 0.01 |
